# A participatory parent-focused intervention promoting physical activity in preschools: design of a cluster-randomized trial

**DOI:** 10.1186/1471-2458-10-49

**Published:** 2010-01-31

**Authors:** Freia De Bock, Joachim E Fischer, Kristina Hoffmann, Herbert Renz-Polster

**Affiliations:** 1 Mannheim Institute of Public Health, Social and Preventive Medicine, University Medicine Mannheim, Heidelberg University, Mannheim, Germany; 2 Department of Pediatrics, University Medicine Mannheim, Mannheim Medical Faculty, Heidelberg University, Mannheim, Germany

## Abstract

**Background:**

With rates of childhood obesity increasing, physical activity (PA) promotion especially in young children has assumed greater importance. Given the limited effectiveness of most interventions to date, new approaches are needed. The General Systems theory suggests that involving parents as intervention targets may be effective in fostering healthier life styles in children. We describe the development of a parent-focused participatory intervention and the procedures used to evaluate its effectiveness in increasing daily PA in preschoolers.

**Methods/Design:**

Thirty-seven South German preschools were identified for this study and agreed to participate. Using a two-armed, controlled cluster-randomized trial design we test a participatory intervention with parents as the primary target group and potential agents of behavioural change. Specifically, the intervention is designed to engage parents in the development, refinement and selection of project ideas to promote PA and in incorporating these ideas into daily routines within the preschool community, consisting of children, teachers and parents. Our study is embedded within an existing state-sponsored programme providing structured gym lessons to preschool children. Thus, child-based PA outcomes from the study arm with the parent-focused intervention and the state-sponsored programme are compared with those from the study arm with the state-sponsored programme alone. The evaluation entails baseline measurements of study outcomes as well as follow-up measurements at 6 and 12 months. Accelerometry measures PA intensity over a period of six days, with the mean over six days used as the primary outcome measure. Secondary outcomes include childrens' BMI, a sum of averaged skin fold thickness measurements across multiple sites, and PA behaviour. Longitudinal multilevel models are used to assess within-subject change and between-group differences in study outcomes, adjusted for covariates at the preschool and individual levels. Teacher qualitative interviews monitor the intervention implementation process.

**Discussion:**

Participatory approaches that actively involve parents have the potential to promote PA in ways that might be better tailored to local needs and more sustainable. Our mixed methods approach to assess the intervention efficacy and implementation employing both quantitative and qualitative measures within a cluster-randomized controlled trial may serve as a framework for evaluating public health interventions in preschool settings.

**Trial Registration:**

clinicaltrials.gov No: NCT00987532

## Background

Over the last 20 years, physical activity (PA) in children has decreased in both developed and developing nations [1]. Lack of PA is a key contributor to the epidemic of childhood obesity and a well-described risk factor for cardiovascular disease [1-4]. In addition to adverse metabolic effects, reduced childhood PA can negatively affect psychosocial factors such as self-esteem [5] and is associated with declining motor skills [6], which may, in turn, contribute further to inactivity. Effective interventions that address childhood PA are therefore urgently needed.

It is not known at what age PA promotion should be instituted to be most effective, but the preschool years may represent a window of opportunity. It is known that 'obesogenic' growth trajectories are often established during the adiposity rebound period [7]. Also, decreased levels of PA in elementary-school aged children are predictive of activity levels later in adulthood [8]. However, few randomized trials evaluating interventions in preschools exist and even fewer interventions have been shown to be effective in reducing overweight and inactivity [9].

Recent research, comprised primarily of studies targeting older children and adolescents, has identified several features that may influence intervention effectiveness [10]. For example, interventions of more than 6 months in duration and those that attempt to reduce inactivity rather than promote vigourous PA [9] may have greater effects. Moreover, efforts to create supportive environments (e.g. playground improvement) [11] and social support (peers, parents) [12-14] have been shown to yield positive results in promoting and sustaining behaviour change in general. Finally, evidence suggests that multi-component interventions may work better than single component interventions in changing health behaviours [15].

For PA interventions in preschool-aged children to be effective, the active involvement of parents may be particularly important. General Systems theory [16] supports this view by suggesting that the involvement of "social players" in a child's life provides role models and establishes patterns of normative behaviour that reinforce behaviour change in children. It is, however, not clear which methods for engaging parents in child PA promotion are most effective [17]. Based on experiences from community-based participatory research [18], a participatory approach including elements of choice, preference and co-determination might foster a sense of self-determination, create greater commitment and eventually lead to greater effects [19]. These features might also foster sustained behaviour changes - a major shortcoming of health promotion interventions that primarily rely upon educational and information-based strategies [20].

While parents were included in some standardized, educational interventions to promote PA in their children (e.g. through homework "assignments", or newsletters) [21-23], the effects of actively involving parents in determining intervention content and structure have not been studied. Their participation in interventions, however, might be essential especially because of their knowledge of the barriers to PA that their children face, but also because they have the best sense for opportunities for improvement that are consistent with their own and their child's preferences [24,25]. Furthermore, parental behaviour is one of the strongest determinants of both child physical activity [26] and Body Mass Index (BMI) [27], which means that involving parents within a participatory framework may foster more active lifestyles in the preschool years and beyond.

To test this hypothesis, we designed a parent-focused, multi-component, participatory intervention to promote childrens' physical activity in preschool settings. Here, we describe the development of the intervention and the methods currently being employed to assess its effectiveness by means of a cluster-randomized controlled trial.

### Theoretical Model

According to General Systems theory [16], health behaviours in children occur within an organic system of preexisting personal relationships and are influenced by a variety of factors including opportunities within the school environment, the home and the community, and the physical characteristics of these settings. Preschoolers' physical activity behaviour is influenced by the attitudes and behaviours of members of their larger network that includes parents, siblings, peer preschoolers and teachers. Although parents assume one of the most influential roles within this network in the preschool years, General Systems theory suggests that efforts to change behaviour should involve as many agents as possible, as behavioural norms develop as a consequence of interactions within the whole network [28].

The design of our parent-focused intervention "Ene mene fit - Eltern machen mit" (translated: Ene mene fit - parents join in) is based upon General Systems theory. The participatory intervention uses parents as the primary target group, but includes the entire preschool community (parents, teachers, children, peers) as potential agents of behavioural change. In order to actively involve the parents, the intervention uses strategies derived from community-based participatory research (CBPR) [18]. Our approach thus relies on the preschool network members' intimate knowledge about locally available resources and barriers to physical activity. This may help tailor the intervention to local needs and resources and may therefore yield greater success and sustainability.

## Methods/Design

### Setting and participants

The study is set in Baden-Württemberg, a federal state in southwest Germany. Its major goal is to increase physical activity in preschools, thus promoting the development of healthy behaviours in young children and decreasing their risk of overweight. With a population of nearly 11 million residents, Baden-Württemberg has an extensive network of more than 7,600 preschools located in urban, suburban, and rural settings, in which three to six year olds are enrolled and cared for by certified teachers. Most children attend preschool for five to seven hours daily.

Because no uniform national guidelines define a minimal frequency for physical activity lessons in preschools, significant variability exists in PA opportunities between preschools, ranging from curricular sport activities to unstructured play activities. To address this, a state-sponsored programme "Komm mit in das gesunde Boot" ("Come aboard the healthy boat") was initiated in 2006 to encourage physical activity among children in Baden-Württemberg. As part of this programme, specially trained external PA teachers deliver 40 standardized one-hour gym lessons over a six-month period (i.e. twice weekly) in preschools that participate in the programme.

**Figure 1:**
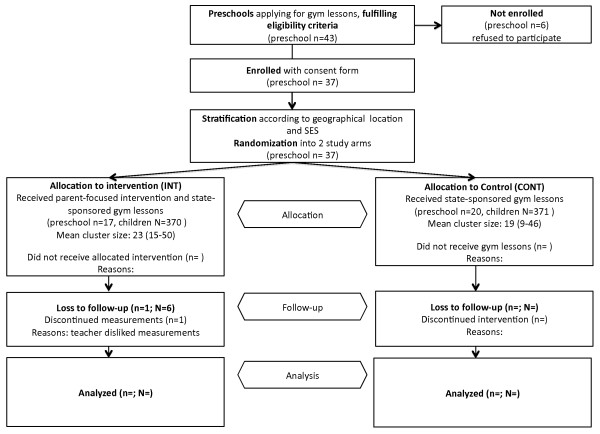
**Study design**. Notes: n = number of preschools, N = number of children, SES = socioeconomic status.

The current study, "Ene mene fit", is a cluster-randomized trial embedded within the state-sponsored programme "Come aboard the healthy boat". It uses a two-level sampling strategy involving both preschools from three geographic regions that had formally applied for participation in the state-sponsored programme and the parents of children enrolled at these sites (see figure 1). Sites were excluded if they had less than 15 children participating in the state-sponsored programme or if there was no external PA teacher available in the area. Parents of any child enrolled at one of the recruited preschools in the intervention arm were potentially eligible for participation in the current parent-focused intervention.

### Intervention components

As a behaviour change strategy, "Ene mene fit" is best described as a complex intervention [29] in which specific components may vary across or within sites, even though each component serves a common purpose or function - to promote routine daily PA in preschool children. Following an extensive analysis of similar projects as well as a literature review, we first generated an initial pool of PA-promoting ideas for use by parents in the intervention arm using a number of techniques. Criteria for incorporating a project idea into the menu of options were that it 1) had already been implemented by parents or preschools in a German speaking community, 2) connected PA with activities of everyday life (e.g., the "walking bus" project for daily transport to preschool), 3) increased time spent outdoors [30] and 4) facilitated PA by building social networks between families. The latter criterion was based upon previous research describing parental lack of time as one of the major obstacles in increasing childrens' PA [25]. This lack of time might be overcome by sharing project responsibilities among several parents within a larger social network.

In a series of pilot tests, the initial pool of project ideas was further refined with input from parents of preschool-aged children. A convenience sample of 22 parents was invited to complete a questionnaire of both closed and open-ended items that assessed potential barriers and preferences for their childrens' PA and the support they might need to foster greater PA in their children. In a subsequent structured telephone interview, we also solicited opinions about the value and feasibility of the project ideas gathered so far as well as about new ideas on other kinds of projects. This process resulted in a menu of 15 easy-to-implement project ideas to promote everyday physical activity in children and families (table 1).

**Table 1:** Excerpts of project idea menu

Evidence-based Principle	Project name	Project description
Active transport	„Walking bus“	Parents organize group walks to preschool (daily schedule)
	
	„Bicycle repair shop“	Parents meet on regular basis to repair bicycles and share skills with children or other parents. Location: either preschool or home garage

Lifestyle physical activity	„Preschool gardening“	Parents and teachers build a garden on the preschool's premises. Teachers involve children in gardening and growing plants.
	
	„Dances from all over the world“	Parents offer regular dance activities during the preschool hours.

Promotion of outdoor activities	„Campfire“	Parents organize regular weekend campfires together with groups of parents and children.
	
	„Forest trip“	Parents organize regular trips to the forest together with teachers during the week or on the weekend.

Reducing obesogenic traditions	„Birthdays and children's parties“	Parents and teachers organize "healthy birthdays" in preschool - Physical activity games and fruit and yoghurt instead of chocolate and cake

With the formal start of the intervention, project ideas were made available to parents on the study website http://www.ene-mene-fit.de and in a printed resource. To promote ease of use, we formatted the presentation of project ideas using four headings: 1) "Project Idea", 2) "Implementation Steps", 3) "Materials Needed", and 4) "More Details and Links". The primary goals in using this format were to provide sufficient details to enable implementation and to provide parents with a structured approach for adding their own project ideas to the website after the end of the formal intervention.

### Intervention structure

As part of the agenda at a regular convocation of parents and teachers, we introduced our intervention using a descriptive video. Following this, parents and teachers were encouraged to discuss barriers and resources for PA within their preschool and generate further ideas on how to enhance the physical activity of their families and among children in their local preschool. Subsequent steps over the course of three follow-up parent-teacher meetings consisted of 1) holding a workshop for project selection and establishing small project-specific teams, 2) team presentations to all parents as a way to motivate a greater number of parents, and 3) a workshop for planning the implementation. Interested parents and teachers were encouraged to identify up to four project ideas that could be taken either from the menu of project ideas or designed *de novo*. We encouraged new ideas, especially if they promoted PA connected to the childrens' everyday life, if they were inexpensive and relatively simple to implement and sustain. To help parents further, we made specially trained physical activity teachers available to offer support by helping to coordinate parent activities (e.g., by proposing time-lines), encourage participation and document the implementation of the project(s).

### Evaluation Design

Our study uses a cluster-randomized trial design, with natural preschool-bound clusters of children randomized to either the intervention or control arm. The evaluation strategy combines both quantitative and qualitative data collection and analyses. Quantitative outcome measurements will be obtained before the start of the intervention (baseline assessment), 6 months after baseline assessment (at the end of the state-sponsored gym lesson programme) and at 12 months after baseline assessment. Qualitative data collection, described in greater detail below, will allow for a closer examination of variation in the implementation process across and within intervention sites. In reporting our study, we will account for the recommendations of the CONSORT statement concerning complex cluster-randomized trials [31,32]. Baseline data from the completed recruitment to the study will be reported elsewhere.

### Recruitment and randomization

**Figure 2:**
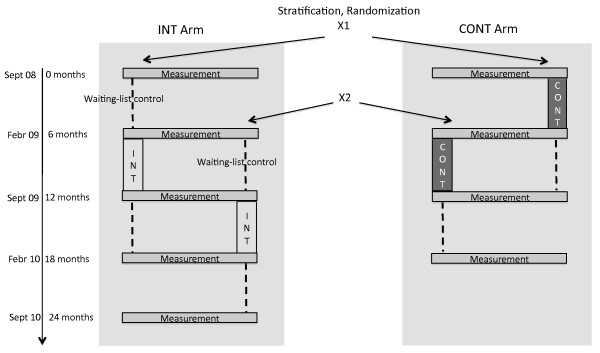
**Evaluation design accounting for seasonal differences and using a waiting list control**. CONT = gym lessons (existing state-sponsored programme). INT = participatory parent-focused intervention plus simultaneous gym lessons. X1 and X2: randomization time points, X1 = autumn, X2 = spring. ---- children enrolled and measured, but no intervention/gym lessons.

Of the 43 preschools applying for participation in the state-sponsored PA programme, we recruited 37 (86%) preschools and a total of 741 (78%) children (see figure 2). As previous research in children has shown differences in PA by physical setting [33] and in BMI by socioeconomic status [27], we stratified the randomization to balance preschool socioeconomic status (low, middle and high) and location (rural or non-rural) in both arms. Aggregate socioeconomic status (SES) of the preschool attendees was estimated by a tertile split based on information provided by the head teacher on the proportion of children from families with low SES or immigrant background. Because we hypothesized that a preschool's location (e.g., amount of green areas, big streets/highways and wooded areas) determined opportunities for PA, we developed a structured protocol to categorize preschools' setting as either rural or non-rural. Specifically, satellite views at a predefined altitude were examined independently by two research team members (Google Earth, accessed 6^th ^June 2008). Rural sites were defined as those that had forest, parks and green spaces within the cutout but no highways or industrial areas. All other preschools were categorized as located in a non-rural area. In each case, ratings were compared and differences were discussed until consensus was reached.

Preschool assignment was blinded through the use of sequentially numbered, sealed envelopes. Preschools in the control arm (CONT) (n = 20; 371 children) receive the state-sponsored programme's gym lessons over six months as the sole intervention. Parents of children enrolled at preschools in the intervention arm (INT) (n = 17; 370 children) receive the participatory intervention, while their children also participate in the gym lessons. The participatory parent-focused intervention runs simultaneously with the gym lessons over the first six months, but continues for a total of at least nine months.

To account for seasonal differences in physical activity [34,35], the study was implemented at two time-points during the year: half of the preschools started the intervention in autumn 2008 with the other half starting in the spring 2009. To account for changes that occur as a consequence of physical maturation in children over time, the participatory intervention arm served as waiting list control during the first six months. Due to the participatory nature of the intervention, it was not possible to blind study participants or intervention providers. However, those involved in outcome assessment will be blinded to intervention assignment.

### Measurements

The unit of analysis are children between three and six years of age enrolled at the participating preschools. Because the evaluation of intervention effectiveness requires application of a lightweight monitor to record PA and heart rate (Actiheart, CamNtech, Cambridge, UK) to the anterior chest wall as described below, children with severe atopic dermatitis, serious physical malformations or disabilities are neither assessed nor contribute data to the evaluation.

#### Physical intensity

We assess physical activity using one-dimensional accelerometry in the vertical plane, measured over six consecutive days including two weekend days (Actiheart, CamNtech, Cambridge, UK). The Actiheart device has been specifically selected for use in this study because its technical validity and reliability has been established [36] and because it allows recording epochs to be set to intervals of 15, 30 or 60 seconds [37]. In our study, we use an epoch setting of 15 seconds to enable detection of rapid changes in movement intensity and short bursts of moderate-to-vigourous physical activity typically exhibited by young children [38,39]. For transformation into an outcome providing a summary measure of the *intensity *of physical activity, accelerometry counts for every 15-second interval during the six-day measurement will be averaged to a single grand mean value for each child.

**Figure 3:**
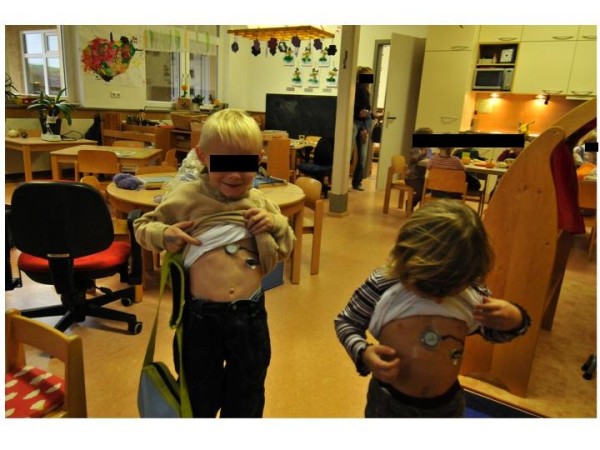
**Preschool children happily presenting their accelerometer**.

According to the measurement protocol, the Actiheart monitors are programmed and securely affixed to each child's substernal thorax [40] by two sticky electrodes (Kendall Arbo*ECG electrodes, Tyco Healthcare, Neustadt Donau, Germany) and additional tape. Subsequently, children participate in a standardized circle-running test for the assessment of heart rate recovery (see below). The monitors stay in place for the next six days; at the end of the recording period, monitors are returned to the research team together with the parental questionnaires in sealed envelopes. In contrast to our expected participation rate of about 60%, almost all children present in the preschool at the measurement day insisted on participating and wearing the device (see figure 3).

#### Anthropometry and body composition

Height is measured to the nearest 0.1 cm (Seca Deutschland, Hamburg, Germany), and weight measured to the nearest 0.1 kg (Soehnle pharo, Nassau, Germany) in underwear following a standardized protocol. Body mass index is calculated using weight (kg) divided by the height squared (m^2^). Instead of using age-specific BMI z-scores, we control for the age-related changes in BMI by inclusion of age as a covariate in the multilevel analyses.

Waist circumference is measured directly on the skin half-way between the top of the iliac crest and the lower rib at the end of gentle expiration [41] with an accuracy of 0.5 cm using a flexible, non-elastic measuring tape (Seca Messband 201, Seca Deutschland, Hamburg, Germany). In addition, waist-to-height-ratio is calculated and serves as a secondary outcome in the analysis of intervention effectiveness [42-44]. Triceps, biceps, subscapular, vertical hip and calf skin folds are measured at least twice using Lange Skin Fold Calipers (Beta Technology, Santa Cruz, USA) with measures averaged for each site. These mean measures will be summed to create an additional secondary outcome - the skin fold sum [45,46]. The quality of the anthropometric measures is ascertained by 1) standardized training sessions for all study personnel conducted over three days and repeated regularly; 2) duplicate measurement of two children per preschool for continuous assessment of interrater reliability and 3) use of a limited number of experienced study personnel to perform skin fold measurements.

#### Parent Questionnaire

A review of previous work assessing health-related behaviours of preschool children by parental proxies revealed few instruments with established psychometric properties. Whenever possible, we use items from existing surveys; in other cases, it was necessary to adapt items to clarify selected features and those specifically related to the intervention. The resulting survey, described below, focuses primarily on children's health behaviours and is completed by one or both of the child's parents. The survey was administered at baseline, six and 12 months and will enable a complementary assessment of intervention effectiveness.

##### Physical activity of the family

Children's PA and sedentary behaviour (e.g., time spent watching television) is being measured using questions from a simple survey instrument that has been validated against a motor skills test [47]. The family's active leisure time is also explored using questions like "How often do you go on excursions (zoo, forest, park, etc.) with your child?" and "How often do you go outside with your child?" Other questions assess parents' leisure time physical activities to establish normative behaviour at baseline. Subsequent assessments will enable an evaluation of potential effects of the participatory intervention on the parents themselves.

##### Nutrition

Childrens' nutrition has the potential to affect most of the secondary outcomes of this study (BMI, skin folds). Therefore, nutrional status is being analyzed as a confounder. Rather than measuring actual caloric intake of the children, however, we focus on the consumption of foods that generally reflect on lifestyle choices. Specifically, we assess the consumption of fruits and vegetables as measured by questions adapted from Bayer et al. [48]: "How many portions (size of a child's hand) of fruit/vegetables does your child eat on average per day?". The consumption of sugared drinks and sweetened or high-caloric snacks is assessed by an adaptation of a food-frequency questionnaire. The food-frequency questionnaire also contains items on a range of indicator foods (fast food, deep-fried) foods, high-caloric food, food especially advertised as healthy for children) and their degree of consumption.

##### Intervention-specific items

Intervention-specific questions evaluate the feasibility of the intervention. For example, to be feasible, the "walking bus" project idea requires a walkable distance between the child's home and the preschool as well as relatively safe traffic conditions. Feasibility is assessed by project-specific items such as "How long does it take you to walk with your child to the preschool?" ("< 5 minutes", "5-10 minutes", "10-15 minutes", "15-20 minutes", "> 20 minutes") and "Are traffic conditions in your neighbourhood safe enough to allow your child to play in the street with other children?" (yes/no).

#### Heart rate recovery

Two-channel electrocardiography using the short-term mode of the Actiheart device (Actiheart, CamNtech, Cambridge, UK) assessed heart rate recovery (HRR), a parameter of cardiovascular fitness and a predictor of mortality in adults [49]. HRR has been traditionally defined by the reduction in heart rate 1 and 3 minutes after a period of submaximal exercise on the treadmill, relative to the peak heart rate at the end of the exercise. In adolescents, HRR is associated with cardiovascular risk factors like serum glucose, triglyceride and CRP levels [50]. Owing to the absence of a valid fitness test in preschool children, an association of HRR with cardiovascular risk or fitness in preschool children has not been previously established[51]. HRR was therefore included for descriptive and exploratory analyses only.

We adapted the traditional measurement protocol for HRR to the field setting in preschools by assessing heart rate after 3 minutes of rest, 2 minutes of circle-running with maximal exertion and again after 3 minutes of rest. The peak heart rate was defined as the highest heart rate in the final 60 seconds of running. HRR is being measured at one and three minutes after the end of exercise. The circle-running test is conducted with at least five and no more than ten children at a time. In a preceding pilot study with 33 preschool children, the latter range was found to be optimal in reducing interferences among children while the test is being conducted.

### Outcomes

#### Quantitative assessment

##### Primary Outcome

• Mean accelerometry counts per 15 seconds over six days of measurement

##### Secondary outcomes

• BMI

• Skin fold sum and central body fat (waist-to-height ratio)

• Physical activity (PA) behaviour via parent questionnaire [47]

##### Other measurements

• Nutritional behaviour, media use

• Heart rate recovery (HRR) after postmaximal exercise

### Research Questions

We aim to show that the supplementation of state-sponsored gym lessons with a parent-focused participatory intervention:

1) leads to significantly increased physical activity intensity in preschool-aged children at 6 months compared with baseline, as measured by accelerometry

2) leads to significant decreases at 6 months compared with baseline in childrens' mean BMI, sum of skin folds and waist-to-height ratio

3) leads to significant improvement in childrens' PA behaviour at 6 months as assessed by questionnaire

4) leads to a significant increase from baseline in childrens' mean 1-minute heart rate recovery after submaximal exercise at 6 months

5) has sustained effects on primary and secondary outcomes stated above (i.e., no significant difference between 6 and 12 months measurements)

Research questions to be addressed using *qualitative techniques *include:

1) How well is the participatory intervention accepted and adopted by parents and teachers?

2) To what extent do participants feel the parent-focused participatory intervention is tailored to the local needs?

3) Do participants feel that the participatory intervention is feasible in the German preschool setting within the framework of an existing state-sponsored programme?

4) What are the barriers and facilitating factors that appear to impede or promote successful intervention implementation and outcomes?

### Covariates/confounders

For correct interpretation of changes in the primary outcome, we are gathering information on factors or characteristics that may affect both the adoption of the intervention [52] and its effectiveness in inducing change. Confounding factors at the child's level include age, gender [53], socioeconomic status (SES) [54] and immigrant background [55]. Sociodemographic information is collected mainly through the parent-completed questionnaire. Immigrant status of each child, for example, is determined by responses to three items: 1) child is a non-German national, 2) child's parents are native speakers of a language other than German 3) child primarily speaks a language other than German at home [56]. Responses (yes = 1; no = 0) were added to create a final value. The measurement of the child's SES is determined from his/her parent's self-report of the highest level of educational attainment [57,58].

The perceived health status of the child and his/her membership in a sports club might moderate the intervention effect [59]. Information on these characteristics therefore is gathered by the following two questions "How would you describe your child's health status?" ("very bad - very good") and "Does your child regularly engage in PA in a sports club or a similar organization?" ("no", "yes, twice monthly", "regularly one hour/week", "regularly two hours per week", "regularly more than two hours per week", "don't know").

### Process measures

We carefully examine the extent to which the intervention is adopted and implemented and whether features of the context in which the intervention is delivered mediate or moderate intervention outcomes. Mediating factors were defined as contextual factors predisposing for or enabling the intended behavioural change, which might lie on the causal pathway from intervention to outcome. Moderating factors were qualitative or quantitative variables that might affect the direction or strength of the relation between intervention and outcome [60]. General Systems theory, for example, suggests the degree of teacher or parent commitment, the co-existence of other preschool programmes and the preschool physical environment as moderating factors. We therefore conduct a thorough process evaluation, which consists of face-to-face, semi-structured key informant interviews with the head teacher at each site and each measurement point. Interviews have been recorded and are currently being transcribed. These data serve as the basis for qualitative analyses to identify emerging issues related to intervention implementation.

#### Measuring the preschool context

The preschool context is assessed by interview questions in order to reveal teachers' expectations of the intervention, perceived level of parental commitment, physical space for recreation (e.g., the availability of outdoor and indoor areas for physical activity), preschool policies concerning physical activity, and existence of concurrent programmes. These data are supplemented by a brief, structured questionnaire to be completed by the head teacher at each preschool on a range of topics including the number of PA promoting toys and daily hours spent outdoors by children at the site. For operationalizing the preschool context as variable, we established a rating system based on the extent to which the preschool allowed and fostered children's PA. Specifically, seven predefined preschool characteristics (e.g. daily time outside, outside space, number of PA promoting toys) will be rated to yield an overall degree of PA facilitation with a possible range from 0 to 53 points for each preschool.

#### Indicators of intervention adoption and implementation

According to the RE-AIM framework [61,62], evaluations of health promotion interventions should not only focus on efficacy, but should include other dimensions of quality like intervention adoption, implementation and maintenance. In our study, the intervention adoption is being assessed by structured interviews with the head teachers before the intervention start and was rated over all preschools on a 1-100% scale. The extent to which the intervention was implemented at each site is assessed by the external PA teacher delivering the state-sponsored gym programme. This external teacher documents numerous features of the implementation process, including the number of project ideas chosen and finally realized, the number of meetings with parents, the participation rate at these meetings, the barriers and resources perceived by parents and teachers, the social environment and development of issues related to effective communication (e.g. atmosphere during meetings, involvement of immigrant parents, coordination between parents and teachers). In addition, information on implementation gathered during the head teacher interviews at each preschool will be used in the analysis. Based on both these data sources, we currently develop definitions for each of the implementation components as well as a ranking system for each component, rated from 1 = ("not realized") to 3 ("completely realized"). Ratings will then be summarized to yield an overall degree of implementation. Intervention maintenance will be explored through interviews with preschool head teachers at 12 months, with data focusing around the extent to which the parents' projects have become an enduring part of the preschool's activity repertoire.

### Assessment of intervention effectiveness

The intervention effectiveness is assessed by an intention-to-treat analysis.

Descriptive statistical analysis will be performed on all outcome measures. If needed, data will be transformed to better meet the assumptions of standard inferential statistical tests. We will use multilevel models to account for the structure of our data and for missing data on any of the measurements. In the multilevel model, repeated measurement of outcomes for each child will be considered the first "level"; these data are nested within preschools, which represent the second level. Differences between intervention and control group in primary and secondary outcomes will be analyzed in terms of fixed effects in the multilevel regression model, with age, sex, SES, immigration status and baseline BMI/accelerometer counts included as covariates on the individual level. In secondary analyses, the effect moderation by the preschool context as rated by qualitative data, as well as the level of implementation and adoption of the intervention will be analyzed.

Previous work documents a higher prevalence of overweight [63] and lower levels of physical activity [64] among immigrants. Because of the potential public health implications, we will conduct subgroup analyses at the child level comparing outcomes for normal-weight versus overweight children and for immigrant versus non-immigrant children. Subgroup analyses at the preschool level will be performed for rural versus non-rural locations and intervention timing (autumn versus spring) to assess the presence of potential effect modifiers.

#### Sample size and power considerations

Previous published work provides little guidance on the extent to which PA-related outcomes in preschool children are inter-correlated. We therefore conservatively estimated the intraclass correlation coefficient (ICC) to be 0.1 ([65], personal communication concerning referenced trial). To detect a difference of 0.5 standard deviations (19.05 counts/15 seconds) between intervention and control group, assuming a standard deviation of 38.1 counts/15 seconds (Corder, K, unpublished data from pilot studies in 4-5 year-old children) for accelerometry and an intraclass correlation of 0.1, with a power of 0.9 and alpha of 0.05 (two-sided), we estimated that a total of 504 children in 24 schools would be needed. We increased recruitment goals to 560 children (280 per arm) to account for a 10% loss to follow-up.

### Qualitative analysis

In addition to the quantitative data on potential determinants of intervention effectiveness, qualitative data could help to reveal factors that may explain baseline and intervention effect differences between preschools. Specifically, we will compare preschools where the intervention is particularly successful and those at which the intervention appeared to fail and will report this using a contrasting case studies approach. Furthermore, only preschools where the intervention was implemented to an acceptable degree (at least one project realized) will be included into the analysis per protocol to grasp the "true" effect of the intervention.

Our qualitative data also serves to explore the acceptance of interventions and to monitor adoption, implementation and maintenance of the parent-focused intervention. Specifically, we will use template analysis to validate previously under-recognized themes we perceive might be related to barriers and resources to the adoption and implementation of the intervention by successive readings and persistent immersion in transcribed text from interviews with head teachers and in documentation generated by the PA teacher [66]. The validity of themes that emerge from this analysis will be tested through an iterative process of review by two independent reviewers who seek refuting or confirmatory evidence in subsequent interview texts.

### Ethics and human subjects' confidentiality

All parents of children participating in the gym lessons received written information on the study and further oral explanation by preschool teachers, if needed. The parents of all children participating in the study provided their informed consent. Ethical approval was granted by the Ethics committee of the Medical Faculty Mannheim, Heidelberg University (ID 2008-275N-MA).

## Discussion

In the present paper we describe the design and evaluation of a public health intervention specifically aiming at promoting preschoolers' PA by involving the individual child's and peers' parents. The study design employing mixed methods to assess the intervention efficacy and implementation within a cluster-randomized controlled trial may serve as a framework for evaluating future public health interventions in preschool settings.

The intervention we describe here has several unique elements. First, we draw from General Systems theory as a conceptual framework for the design of our intervention. This theory suggests, for example, that participation of the whole preschool social network (parents, children, grandparents and teachers) may allow for a more customized approach, which in turn may increase the acceptance and effectiveness of the intervention. This theory also suggests that the social network developing among participants of a participatory intervention may lead to longer-term sustainability. To enable network building among participants, we use readily available website authoring technology to create a shared information platform. This feature also has the potential to sustain effects of the intervention following the end of the formal study intervention by providing a no-cost resource to a broad community of parents and teachers with common interests. Second, our intervention moves beyond a knowledge-based or educational approach by fostering "learning by doing" within child and parent peer groups. Third, we have designed and implemented a multi-component intervention that incorporates an existing state-sponsored programme to promote PA. As a fourth element we use a multi-pronged strategy to change behaviours. In many interventions, for example, the starting point of PA promotion is sports. We chose to broaden our focus by including lifestyle-related activities (e.g. active transportation) that could be practiced daily. We also deliberately included non-athletic forms of PA (e.g. dancing, theatre), which may appeal to a broader part of the preschool children. Offering multiple entry points to PA may especially help to develop gender-specific approaches for PA promotion and may prove to be a more inclusive strategy, which may especially benefit immigrant children. Finally, our mixed methods evaluation strategy will enable insights into factors serving as barriers or facilitators to successful implementation of the intervention, thus strengthening the design of subsequent work in a relatively understudied but highly important population. Because a participatory approach has the potential to reveal unanticipated solutions as well as unexpected challenges that threaten intervention implementation and effectiveness, it is important that all participants including the research team use qualitative methods to enable learning.

Despite its strengths, we acknowledge several limitations of our study design. Our intervention does not target all levels of the system, in which preschoolers' behaviours develop. For example, the final menu of project ideas has not been developed in the teachers' environment and is not anchored within the curricular framework of the preschool teachers. Previous participatory research suggests that this might hamper the readiness of teachers to take ownership in the intervention change process [67]. Second, while stakeholders of the preschool communities participate in contents and structure, they are not included into the planning of the intervention. Therefore, our study cannot be characterized as community-based participatory research in a literal sense. However, a recent systematic review of community-based participatory research (CBPR) found only 4 of 60 CBPR studies demonstrating community participation across all research phases [68]. A further limitation of our participatory intervention is that the initial set of ideas was not refined and discussed with input from parents of children enrolled at the study preschools, but rather from parents obtained through a convenience sample of preschools prior to study inception. We will therefore explicitly check for differences in the discussions on the project ideas between the parents from the convenience-sampled versus the study preschools by means of qualitative data.

Although we were interested in using methods that promoted intervention sustainability, several factors may work against this. Our intervention, as currently proposed, will require some degree of parental time commitment at a level that might exceed parental resources. This may, in turn, threaten sustainability through fluctuations in parental time availability. In addition, changes in parental involvement may be expected as children graduate from kindergarten. To limit potential effects on the change process, we will seek ideas on how to minimize the knowledge and leadership drain, e.g. by encouraging effective documentation or by giving helpful hints to subsequent groups of parents in the last parent meeting during the formal intervention period. We anticipate that this sort of "sustainability planning" will be facilitated by placing implementation resources and project ideas on the study-dedicated website. Despite these efforts, greater reliance on teachers rather than parents may be required to successfully implement programmes that promote the adoption and routine practice of healthy behaviours.

As in any preventive intervention, the ultimate success hinges on building "capacity" - an intervention is only effective in the long run if it enables individuals or organizations to incorporate the goals of the intervention into their routine daily activities. The theory-based participatory approach used in this study can be conducive to capacity building by fostering a "pro PA" culture within the local preschool setting and providing resources to sustain new normative behaviours. Enriching children's environments with opportunities for PA may therefore become a normative expectation within the preschool community transmitted from the parents of one preschool class to the next. The effective implementation of participatory PA interventions in preschools might be one strategic element in lowering the public health burden of physical inactivity in younger children. Our study design, which employed a cluster randomized controlled design including a waiting-list and sought to avoid randomization imbalances by stratifying preschools according to average social class and to rural vs. non-rural natural setting, may serve as a role model for evaluating public health interventions in the preschool context.

## Competing interests

The authors declare that they have no competing interests.

## Authors' contributions

FDB: first author, main writer of manuscript, substantial contributions to conception and design, acquisition of data and plan of analysis. HRP: substantial contributions to conception and design, acquisition of data and plan of analysis, internal reviewer of manuscript. JF and KH: substantial contributions to conception and design, given final approval of the version to be published. All authors read and approved the final manuscript.

## Pre-publication history

The pre-publication history for this paper can be accessed here:

http://www.biomedcentral.com/1471-2458/10/49/prepub
