# Correction: A participatory parent-focused intervention promoting physical activity in preschools: Design of a cluster-randomized trial

**DOI:** 10.1186/1471-2458-12-355

**Published:** 2012-05-16

**Authors:** Freia De Bock, Joachim E Fischer, Kristina Hoffmann, Herbert Renz-Polster

**Affiliations:** 1 Mannheim Institute of Public Health, Social and Preventive Medicine, University Medicine Mannheim, Heidelberg University, Mannheim, Germany; 2 Department of Pediatrics, University Medicine Mannheim, Mannheim Medical Faculty, Heidelberg University, Mannheim, Germany

## Correction

At the time of writing our study design paper [1], the study's recruitment and randomization process was ongoing with numbers of schools in both study arms still changing. We finally were able to recruit more eligible preschools than initially thought. Therefore the numbers in Figure 1 have changed (see new attached Figure 1).

**Figure 1:**
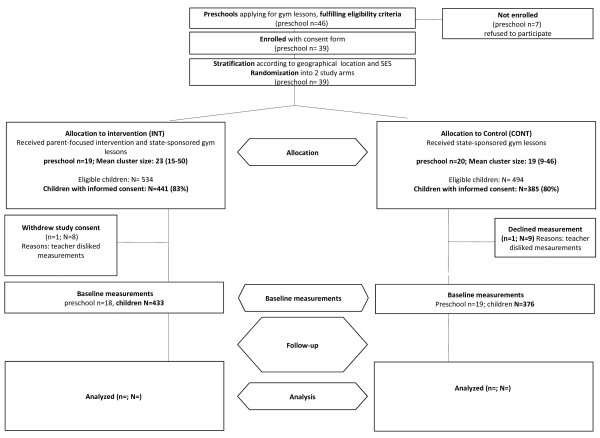
Study design notes: n = number of preschools, N = number of chlidren, PA (physical activity), Measurement: accelerometry and heart rate measurement over 6 days, SES = aggregate socioeconomic status.

Of finally 46 eligible preschools applying for participation in the state-sponsored PA programme, we recruited 39 (86%) preschools and a total of 826 (80% of eligible) children. Nineteen and 20 preschools (with 441 and 385 children with informed consent) were randomly assigned to the intervention and control group, respectively, after stratification for aggregate SES [1] and geographical location. One preschool per arm left the study before baseline, leaving 433 children in 18 intervention and 376 children in 19 control preschools for analysis.

In the ethical approval, we announced a minimal number (based upon sample size calculations): 280 per arm = 560 total, but had permission from the ethics committee to recruit additional participants in preschools.

The increase in preschool and participant numbers might enable us to grasp smaller clinical differences than originally planned. However, consideration of the revised figure with respect to participant flow does not result in any qualitative change of the conclusions of our previous report.

We regret any inconvenience that this inaccuracy due to the publication of preliminary numbers of eligible and recruited preschools might have caused.

## Pre-publication history

The pre-publication history for this paper can be accessed here:

http://www.biomedcentral.com/1471-2458/12/355/prepub
